# A synthetic analysis of greenhouse gas emissions from manure amended agricultural soils in China

**DOI:** 10.1038/s41598-017-07793-6

**Published:** 2017-08-14

**Authors:** Fengling Ren, Xubo Zhang, Jian Liu, Nan Sun, Lianhai Wu, Zhongfang Li, Minggang Xu

**Affiliations:** 1 Institute of Agricultural Resources and Regional Planning, Chinese Academy of Agricultural Sciences/National Engineering Laboratory for Improving Quality of Arable Land, Beijing, 100081 China; 2 Key Lab. of Ecosystem Network Observation and Modeling, Institute of Geographic Sciences and Natural Resources Research, Chinese Academy of Sciences, Beijing, 100101 China; 3 Department of Plant Science, Pennsylvania State University, University Park, Pennsylvania, 16802 USA; 4 Sustainable Soils and Grassland Systems Department, Rothamsted Research, North Wyke, Okehampton, Devon, EX20 2SB UK; 5 Chemistry and Bioengineering College, Hezhou University, Hezhou, 542899 China

## Abstract

Application of manure has been recommended as an effective strategy to to mitigate climate change. However, the magnitude of greenhouse gases emission derived by application of manure to agricultural soils across environmental conditions still remains unclear. Here, we synthesized data from 379 observations in China and quantified the responses of soil nitrous oxide (N_2_O), carbon dioxide (CO_2_) and methane (CH_4_) emissions to manure (Org-M) in comparison to chemical fertilizers (Min-F) or non-fertilizers (Non-F). The results showed that N_2_O, CO_2_ and CH_4_ emissions were significantly affected by Org-M compared to Min-F (percentage change: −3, +15 and +60%, *P* < 0.05) and Non-F (percentage change: +289, +84 and +83%, *P* < 0.05), respectively. However, at the same amount of total N input, Org-M decreased soil N_2_O emission by 13% and CH_4_ emission by 12%, and increased soil CO_2_ emission by 26% relative to Min-F in upland soils. For paddy soils, N_2_O, CO_2_ and CH_4_ emissions differed by −3%, −36% and +84% between Org-M and Min-F (i.e., Org-M minus Min-F). Thus, practices such as application of manure instead of chemical fertilizer and decreasing nitrogen input rate need to be highly considered and optimized under different soils and climate conditions to mitigate GHGs emission in China.

## Introduction

Greenhouse gases (N_2_O, CO_2_ and CH_4_) emitted from agricultural soils have been recognized as a major contributor to global warming. It has been estimated that more than 13% of the global anthropogenic GHGs including 60% of CH_4_ and N_2_O are associated with direct soil-derived GHGs and agricultural inputs^1–5^. Agricultural soils may become a net source or sink of GHGs depending on different management strategies such as application of chemical fertilizers (Min-F) or manure (Org-M)^6^. In addition, the emissions of GHGs can also be altered by changes of the amount and chemical compositions of manure applied to soils^7^.

Soil N_2_O is the production of microbial processes via denitrification and nitrification under dry and wet conditions^8, 9^. Emission of N_2_O can present high spatial and temporal variability^10^, and it can be dramatically changed by farming practices such as fertilization^11, 12^. For example, Bouwman *et al*.^11^ and Stalenga and Kawalec^13^ reported that application of chemical N fertilizer resulted in soil N_2_O emission nearly two-fold higher than that by application of animal manure.

Soil CO_2_ emission in agricultural soils is derived from rhizosphere respiration and soil microbial respiration^14^. In general, cumulative CO_2_ emission was significantly correlated with soil organic carbon (SOC) content because the amount of substrates for soil microorganisms can be greatly increased by SOC and soil microbial activity can be further altered^15, 16^. Recently, it was found that organic farming or animal manure application can potentially sequestrate more C to the soils and thus convert the soils to a net CO_2_ sink^17–19^. However, some previous studies presented different or opposite response of soil CO_2_ emission to Org-M under different conditions. For instance, it was reported that cumulative soil CO_2_ emission in the manure treatments was lower than that in chemical fertilizer treatments of conventional farming in upland soils^16, 20^, but the others also reported that manure did not accelerate soil CO_2_ emission in upland soils even though SOC was sharply increased^14, 21–24^. The inconsistent results indicated that the response of soil CO_2_ emission to manure application was dependent on environmental factors such as climate and soil properties and management factors such as land use and fertilization^25, 26^. Thus, quantifying the influence of these factors in solving uncertainties regarding spatial and temporal variation in soil CO_2_ emission related to manure application is highly needed^27^.

Soil CH_4_ is produced when organic matter is decomposed and CO_2_ is reduced under highly anaerobic environments^28^. And waterlogged rice paddies are a major source of CH_4_ emission^29^. By contrast, well-aerated or drained arable land is usually a sink for atmospheric CH_4_, because CH_4_ can be used by soil methanotrophs as a source of carbon and energy^28, 30^. However, well-aerated agricultural soils can shift to CH_4_ sources for a certain period of time when excessive amount of manure with high organic matter content is applied to the soil^31^. Thus, the effect of application of manure on reducing CH_4_ emission and improving carbon sequestration need to be further clarified under waterlogged rice paddies or well-aerated or drained upland soils.

China’s agriculture is facing a crucial challenge of ongoing environmental degradation and to ensure food security^32^. Thus, it is very urgent and necessary to improve farming practices and cropping techniques for sustaining high yield level while mitigating GHGs emissions. In addition, there are very rare studies that have addressed the effect of manure application on GHGs emissions with a comparison to non-fertilizer and chemical fertilizers in China^33^, even though organic farming practices can potentially mitigate GHGs emissions^29^. Over the past decades, published studies from China were mostly focused on the impact of specific farming practices on GHGs emissions at a field or a regional scale, which can hardly present a complete perspective on the effects of manure application on GHGs emissions across China’s major grain production regions.

Meta-analysis has emerged as a very useful approach to quantitatively synthesize, analyze and summarize the results of a collection of studies^34^. The analysis method offers a formal statistical analysis to integrate and compare the results collected from multiple studies and to draw general patterns at various spatial scales, and the outcomes of published studies are treated as if they are subject to sampling uncertainties^34–36^. Therefore, it has been used to analyze GHGs emissions at national or global scales^2, 5, 37–39^. Here, a meta-analysis was conducted to systematically compare the soil GHGs emissions under Org-M to Min-F and Non-F systems under different land uses, climate types, soil pH, soil total nitrogen (TN), soil organic matter (SOM), total N input in China. The objectives of the current study were: 1) to quantitatively assess the magnitude of manure application impacts on GHGs emissions compared with application of chemical fertilizers and non-fertilized system, and 2) to quantify the effects of manure application on GHGs emissions under different conditions including climate, land use, soil pH, soil nitrogen (N) content and soil organic matter level.

## Materials and Methods

### Data sources and selection

To fully cover the published research on assessing greenhouse gas emissions from Chinese soils, a total of 1500 peer-reviewed articles indexed by the Web of Science (http://apps.webofknowledge.com/) and the China Knowledge Resource Integrated Database (http://www.cnki.net/) were retrieved for the period from 1900 to 2016. The keywords of manure sources (animal, pig, cattle, hog, poultry, sheep, horse, compost, manure, dung, farmyard manure, etc.), and greenhouse gases (GHGs, nitrous oxide, carbon dioxide and methane fluxes) were used in the literature retrieval. This study aimed at evaluating responses of GHGs emissions to manure application in comparison to non-fertilizers and chemical fertilizers, for which a total of 90 articles were selected for the meta-analysis. Specifically, the following criteria were used to select the publications: 1) field experiments were carried out on crop land in China; 2) there were at least three replications for each treatment; 3) both the treatment with manure application and either the Non-F treatment or Min-F treatment were included in the experiments; and 4) total N inputs were presented or could be calculated.

Firstly, we evaluated responses of GHGs emissions to manure applications in comparison to non-fertilizers and chemical fertilizers (Org-M vs. Non-F and Org-M vs. Min-F separately). Thereafter, the Org-M treatments were further separated to two categories: manure alone (OM) or chemical N, phosphorus (P) and potassium (K) plus OM (OM + CF). This separation was based on the fact that manure could be applied alone or combined with mineral fertilizers in farmers’ practices, and it could carry the same amount of N or additional amounts of N, P, and K compared to Min-F treatments in the same study. In addition, the total N inputs from different studies were further separated as ‘different’ or ‘same’ amount of total N input for the treatments of chemical N only, manure only or chemical N plus manure to analyze the influences of Org-M on GHG emissions under same amount of N input. Information on mean, standard deviations (or standard errors), and magnitude of seasonal cumulative emissions of N_2_O, CO_2_ and CH_4_ was either available in the publication or could be calculated. Cumulative emissions (kg ha^−1^) of soil N_2_O, CO_2_ and CH_4_ during a crop growing season were collated in the dataset for each study.

Among the 90 publications selected for the synthesis analysis, 85 were related to N_2_O emission (57 for upland soils and 28 for rice paddies), 67 to CO_2_ emission (44 for upland soils and 27 for rice paddies) and 42 to CH_4_ emission (10 upland soils and 32 rice paddies) (see Appendix Table 1). The soil depth considered was 0–20 cm. For each original study, the following information was compiled into the dataset: experimental location (longitude and latitude), duration of the experiment, soil acidity and alkalinity (pH), soil organic matter content and soil TN content at the start of the experiment, land use (rice paddies or upland soils), crop species, input rate of N in chemical and manure treatments.

### Data preparation

The data from the studies, which provided the cumulative N_2_O, CO_2_ and CH_4_ emissions (kg C or N ha^−1^) during wheat, maize and rice growing reasons using static chamber method, were collected. Meta-analysis was also used to determine changes in soil GHGs emissions after application of manure to soils in various soil and environmental conditions. Means (M), standard deviations (SD), and sample sizes (n) of the selected variables were extracted from publications for each case study. If only the standard errors (SE) were given in a paper, SD was calculated by:

1 $${\rm{SD}}={\rm{S}}{\rm{E}}\sqrt{{\rm{n}}}$$

A natural log-transformed response ratio (ln*RR*) was employed to reflect the effects of manure application on gas fluxes, and calculated by Hedges *et al*.^35^:

2 $$\mathrm{ln}\,RR=\,\mathrm{ln}({\bar{x}}_{t}/{\bar{x}}_{c})=\,\mathrm{ln}({\bar{x}}_{t})-\,\mathrm{ln}({\bar{x}}_{c})$$

where the subscript of *t* and *c* represents treatment and control, respectively; and $\bar{x}$ is a mean of variable *x* either for a treatment or control.

In addition, the weighing factor (*w*_*ij*_), weighted response ratio (*RR*_++_), the standard error of (*RR*_++_) (S), and 95% confidence interval (CI) of (*RR*_++_) were calculated as below^37, 40^:

3 $${w}_{ij}=\frac{1}{v}$$

4 $$v=\frac{S{D}_{t}^{2}}{{n}_{t}{\bar{x}}_{t}^{2}}+\frac{S{D}_{c}^{2}}{{n}_{c}{\bar{x}}_{c}^{2}}$$

5 $$R{R}_{++}=\frac{\sum _{i=1}^{{\rm{m}}}\sum _{j=1}^{ki}{w}_{ij}R{R}_{ij}}{\sum _{i=1}^{{\rm{m}}}\sum _{j=1}^{ki}{w}_{ij}}$$

6 $$S(R{R}_{++})=\sqrt{\frac{1}{\sum _{i=1}^{{\rm{m}}}\sum _{j=1}^{ki}{w}_{ij}}}$$

7 $$95 \% CI=R{R}_{++}\pm 1.96S(R{R}_{++})$$

where *n*_*t*_ and *n*_*c*_ are number of samples in a treatment and reference control, and the SD_t_ and SD_c_ are standard deviation of a treatment and reference control, respectively.

If the 95% CI of cumulative N_2_O, CO_2_ and CH_4_ emissions did not overlap with zero, the treatments were considered to represent a significant increase (>0) or decrease (<0) compared to the controls of those two variables (P < 0.05). But if it overlapped with zero, the response of that variable to manure application represented no significant difference with Min-F or Non-F^41^. The percentage of change in N_2_O, CO_2_ and CH_4_ cumulative emissions from Org-M compared with Non-F and Min-F was calculated by the equation of (e^*RR*++^−1) ×100%, which the equation has been used previously^5, 37^.

Frequency distributions of ln*RR* were plotted to reflect the variability of manure application effects among different studies by a Gaussian function (i.e., normal distribution)^37^:

8 $${\rm{y}}={\rm{\alpha }}{e}^{\frac{{(x-\mu )}^{2}}{2{\sigma }^{2}}}$$

where y is the frequency of ln *RR* values within an interval, *x* is the mean of ln*RR* for that interval, *μ* and *σ* ^2^ are the mean and variance of all ln*RR* values, respectively, and ${\rm{\alpha }}$ is a coefficient indicating the expected number of ln*RR* at *x* = *μ*.

### Statistical analysis

The METAWIN 2.1 software was employed for meta-analysis^42^. Different categorical variables were used to examine the effect sizes of the comparisons of various conditions that introduced above: land use type, climate types, soil pH, TN, SOM, total N input. Among these variables, due to the large differences of soil N_2_O, CO_2_ and CH_4_ emissions between rice paddies and upland soils, the Org-M comparisons effects in the meta-analysis were divided into two categories: rice paddies and upland soils. Furthermore, to test the Org-M impacts on CH_4_ uptake in upland soils, only the negative values comparisons of emission were extracted and shifted to positive values for meta-analysis^5^.

Additionally, the seven categorical variables (land use type, climate types, soil pH, experimental SOM and TN content, total N input) were all analyzed in the calculation of effect sizes and comprehensive assessment of soil N_2_O, CO_2_ and CH_4_ emissions. Land use types were classified into two categories: rice in paddy soils and crops in upland with or without irrigation^43^. Three major climate types were dominated in agricultural soils in China: temperate monsoon climate (NTM), temperate continental climate (NTC) and subtropical monsoon climate (STM). Soil pH was classified into two categories: pH < 7 (acid soils) and >7 (alkaline soils)^5^. Four levels of SOM were used: <10.0 (poor), 10.0–21.0 (less), 21.0–35.0 (medium) and >35.0 (rich) g DM/kg soil. Soil TN were also divided into four groups: <0.5 (poor), 0.5–1.0 (less), 1.0–2.0 (medium) and >2.0 (rich) g N/kg soil. In the treatments of Min-F and Org-M, both the amount of total N input is equal or not which has large difference effect on the GHGs emissions. According to the amount of total nitrogen input, GHGs emissions in Org-M was compared to Min-F at the same amount of total N input. SigmaPlot 11.0 (Systat Software, San Jose, CA) was used to fit data to normal distribution.

## Results

### Responses of GHGs emissions to Org-M

**Figure 1:**
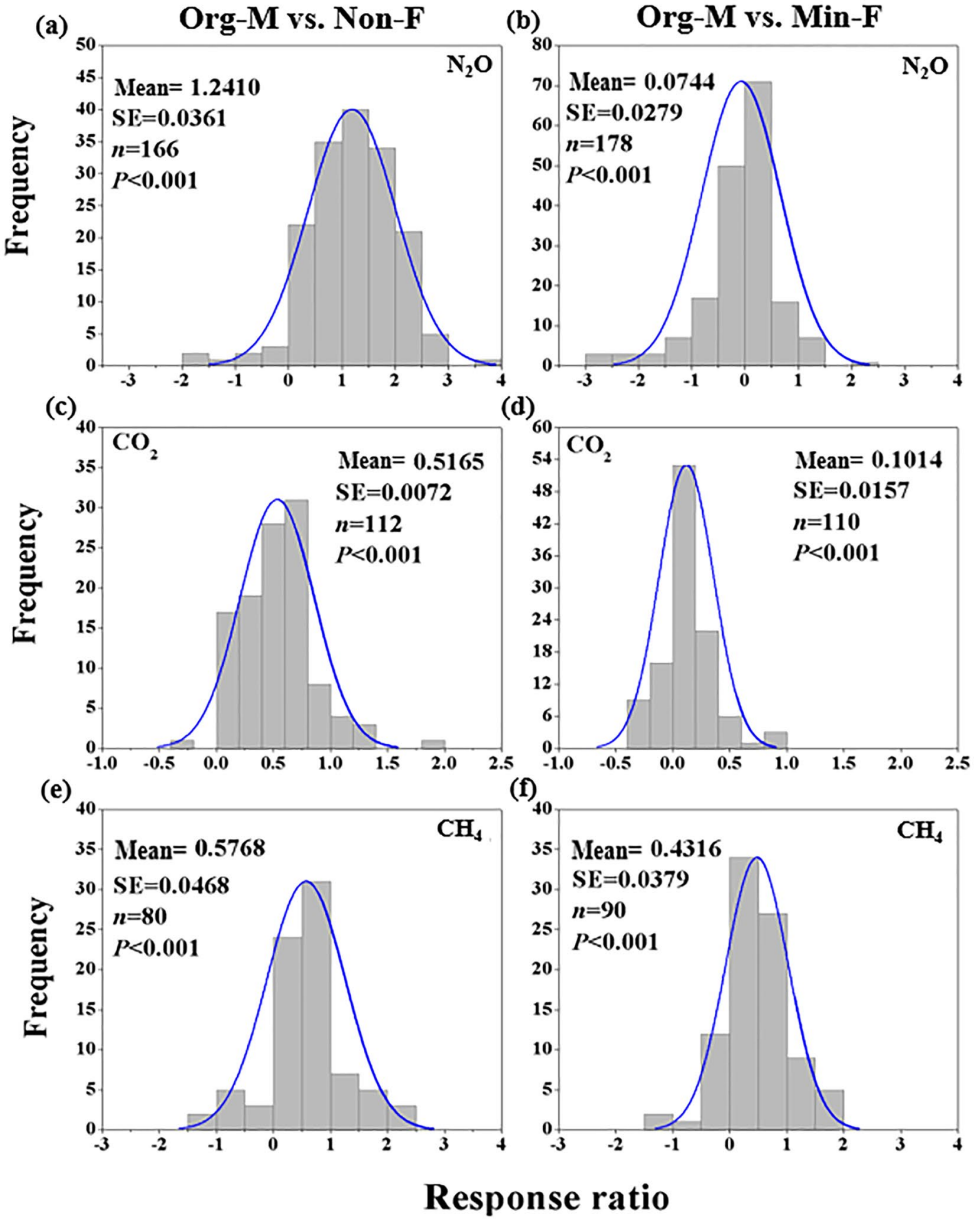
Frequency distributions of response ratios (ln*RR*) for N_2_O (**a**,**b**), CO_2_, (**c**,**d**) and CH_4_ (**e**,**f**) responses to Org-M in comparison with the control group Non-F and Min-F, respectively. The solid curve is a Gaussian distribution fitted to frequency data.

Response ratios for soil N_2_O, CO_2_ and CH_4_ emissions to Org-M relative to Non-F and Min-F were shown in Fig. 1, where comparisons between Org-M and Non-F had overall greater response ratios than comparisons between Org-M and Min-F. In comparison with the group of Non-F, Org-M significantly increased N_2_O, CO_2_ and CH_4_ fluxes, with a mean value of 1.24 ± 0.036 (mean ± 95% CI, same for below) for N_2_O, 0.517 ± 0.072 for CO_2_ and 0.577 ± 0.047 for CH_4_ (Fig. 1a,c and e), i.e., an increase by 289%, 84% and 83%, respectively. Furthermore, in comparison with Min-F, manure application led to an increase in C and N fluxes with a mean of 0.074 ± 0.028 for N_2_O, 0.101 ± 0.016 for CO_2_ and 0.432 ± 0.038 for CH_4_ (*P* < 0.001; Fig. 1b,d and f), i.e., an increase of −3%, 15% and 60%, respectively.

### Meta-analysis results on GHGs emissions

**Figure 2:**
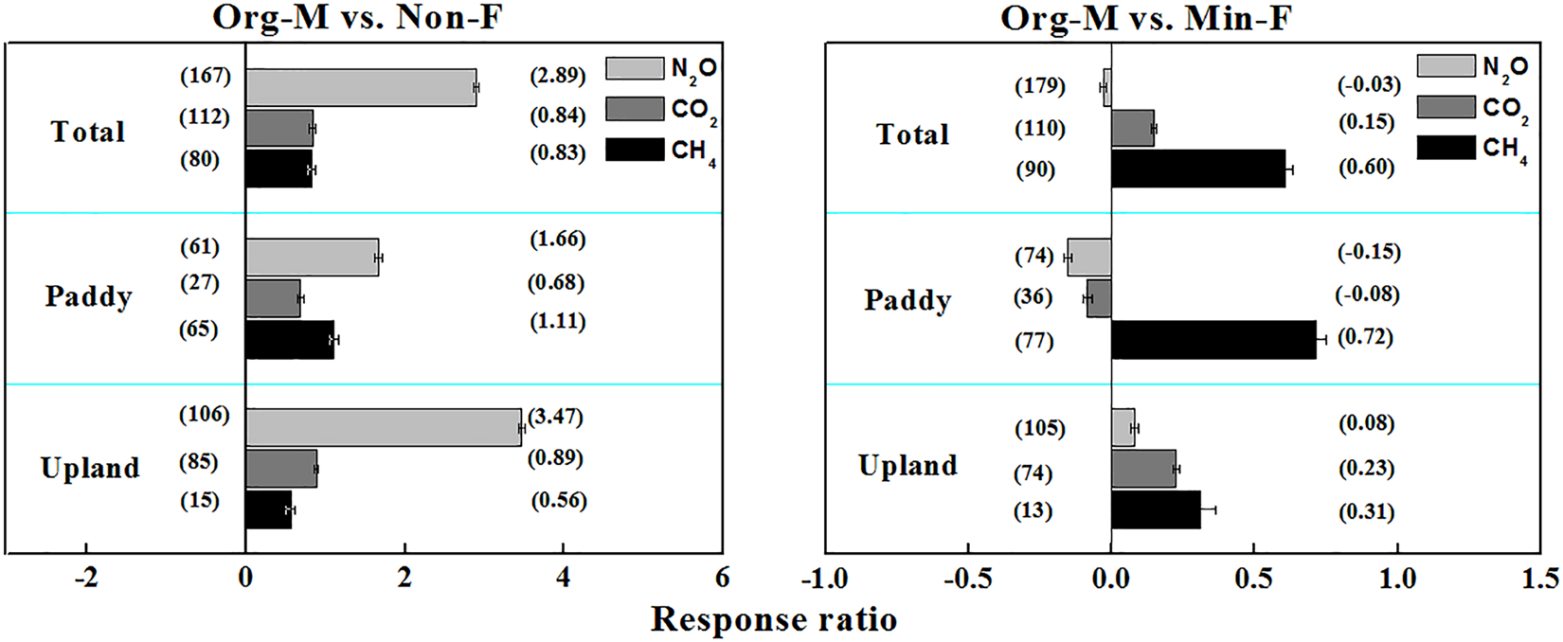
N_2_O, CO_2_ and CH_4_ emissions (CH_4_ emission for paddies and uptake for upland) affected by manure application compared to Non-F (left panel) and Min-F (right panel). Numbers near the bars at the positive side of x axis are the *RR*_++_ and the numbers at the negative side of x axis are the numbers of comparisons. *P* < 0.05, when error bars do not overlap zero.

The group analysis showed that manure application consistently increased soil N_2_O, CO_2_ and CH_4_ emissions compared to Non-F, while the effect of Org-M in comparison to Min-F was greatly affected by land uses, i.e., upland soils and paddy soils (Fig. 2). Specifically, compared to Non-F, Org-M significantly increased N_2_O and CO_2_ emissions by an average of 289% and 84% (*P* < 0.05), with an increase of 166% and 68% in paddy soils, and 347% and 89% in upland soils (Fig. 2), respectively. In addition, compared to Min-F, manure application decreased soil CO_2_ emission by 8% in paddy soils but increased CO_2_ emission by 23% in upland soils (*P* < 0.05; Fig. 2). Similarly, Org-M reduced N_2_O emission by 15% in paddy soils but increased the emission by 8% in upland soils.

**Figure 3:**
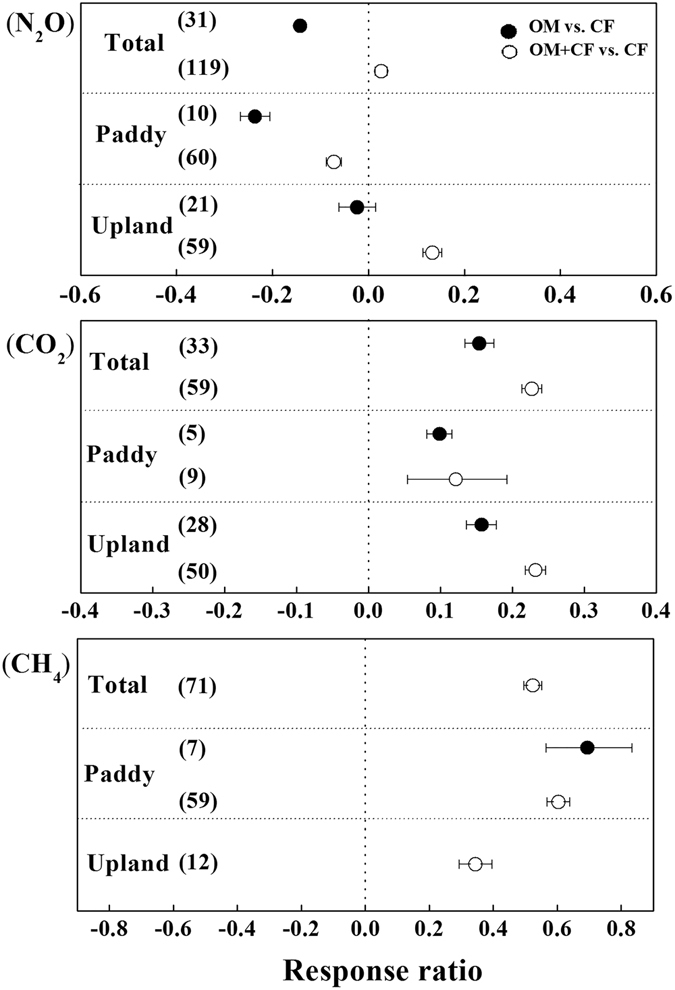
N_2_O, CO_2_ and CH_4_ emissions (CH_4_ emission for paddies and uptake for upland) affected by manure application (OM + CF and OM) compared to Min-F respectively. *P* < 0.05, when error bars do not overlap zero.

**Figure 4:**
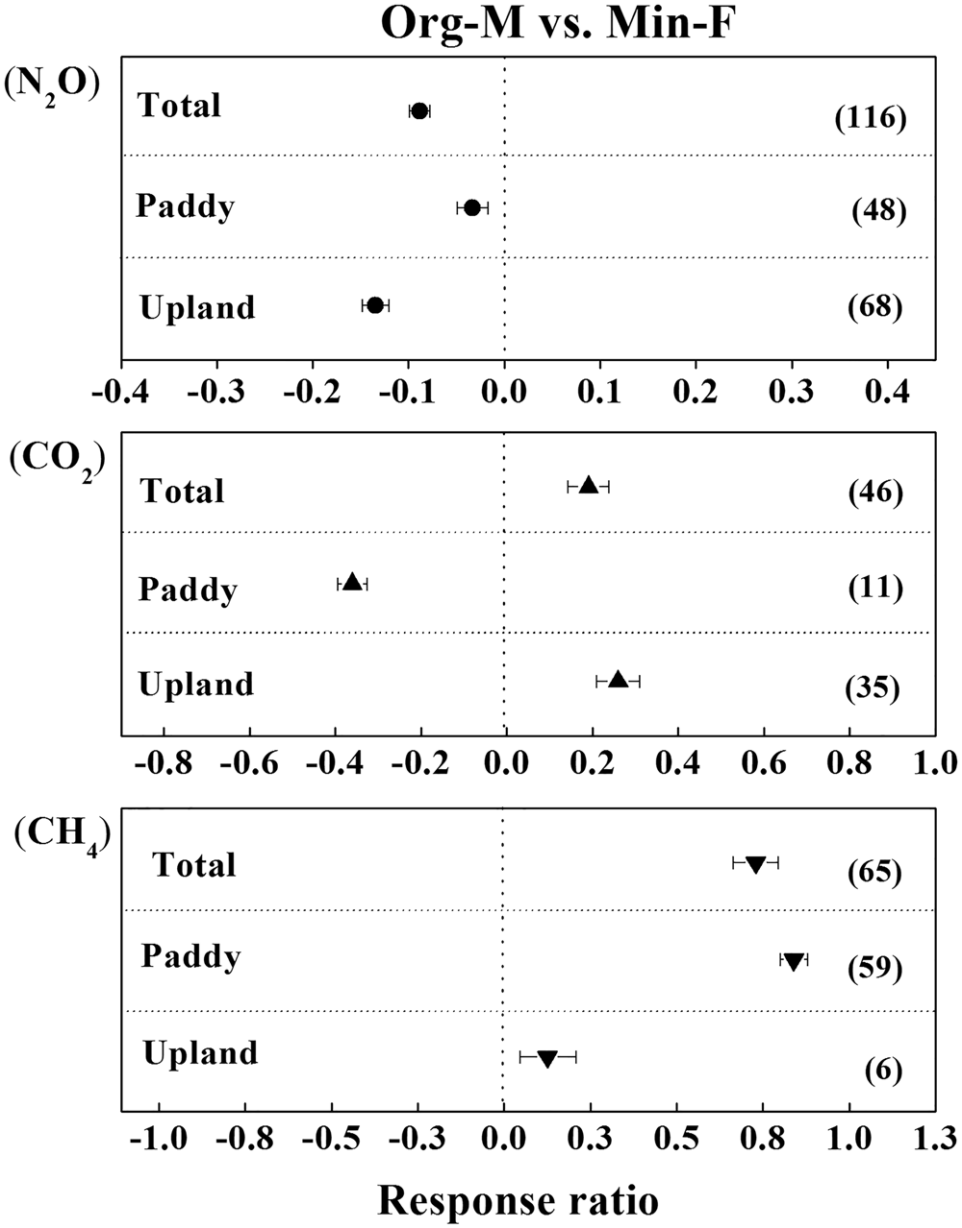
N_2_O, CO_2_ and CH_4_ emissions (CH_4_ emission for paddies and uptake for upland) affected by manure application compared to chemical fertilizers with same amount of N input. *P* < 0.05, when error bars do not overlap zero.

The magnitude of GHGs emissions from the soils with OM and OM + CF compared to Non-F and Min-F was shown in Fig. 3. Over all land uses, N_2_O emission from the soils with OM were 14% less than those from Min-F, but the emission in OM + CF were 3% higher than that in Min-F (Fig. 3). Specifically for different land uses, both OM and OM + CF decreased N_2_O emission in paddy soils compared with Min-F (by 24% and 7%, respectively), while in upland soils OM decreased but OM + CF increased soil N_2_O emission. Furthermore, it should be noted that soil N_2_O emission from Org-M was 9% lower than that from Min-F when the total N input to the soil was same (Fig. 4).

Differing from that for N_2_O, OM and OM + CF consistently increased CO_2_ emission in both paddy and upland soils compared to Min-F (Fig. 3; *P* < 0.05). Overall, CO_2_ emission was increased by 15% in OM and by 23% in OM + CF. However, at the same amount of total N input, soil CO_2_ emission from Org-M was 36% lower than that from Min-F in paddy soils, but soil CO_2_ emission from Org-M was 26% higher than that from Min-F in upland soils (Fig. 4), which indicates that mitigation of CO_2_ emission in paddy soils could be achieved by reducing manure application rate to a reasonable level.

Behavior of CH_4_ in soil was greatly affected by land use. CH_4_ uptake (negative values) was observed in upland soils, but CH_4_ emission (positive values) was observed in paddy soils. Thus, both the terms of “CH_4_ emission” and “CH_4_ uptake” were used in the meta-analysis. In paddy soils, Org-M significantly increased CH_4_ emission by 111% and 72% in comparison with Non-F and Min-F, respectively (Fig. 2). In addition, CH_4_ emission in OM is 9% greater than in OM + CF compared to Min-F (Fig. 3). Notably, at the same amount of total N input, Org-M increased 84% of CH_4_ emission compared to Min-F, and was the highest among the treatments (Fig. 4).

In upland soils, compared to Non-F and Min-F, Org-M significantly increased CH_4_ uptake by 56% and 31%, respectively (Fig. 2). At the same amount of total N input, Org-M increased CH_4_ uptake by 12% compared to Min-F (Fig. 4). In the current dataset, OM + CF increased the soil CH_4_ uptake by 34% compared to Min-F, while the effect of OM was not analyzed due to insufficient data (Fig. 3).

### Factors affecting the GHGs emission changes

#### N_2_O emission

**Figure 5:**
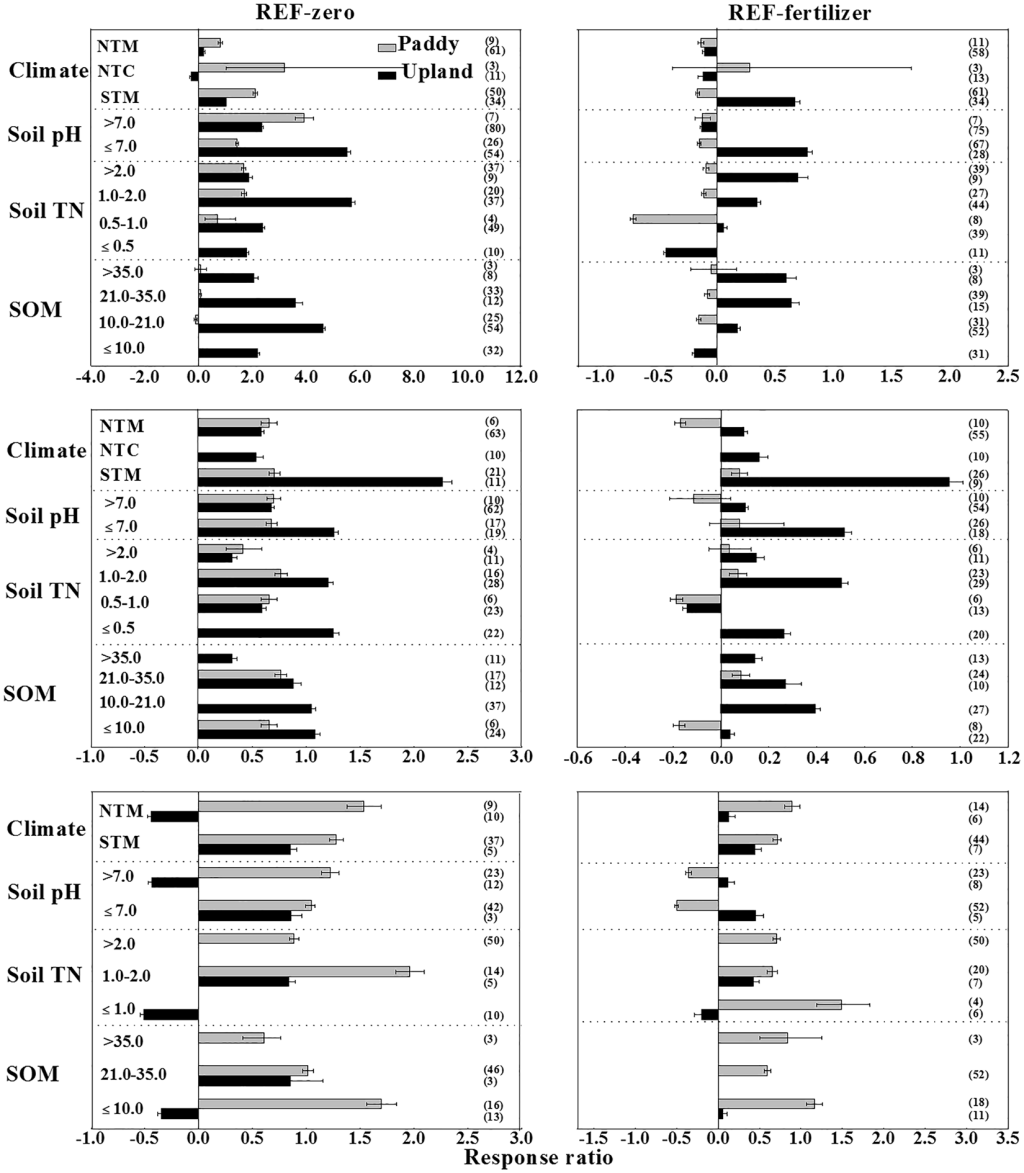
N_2_O, CO_2_ and CH_4_ emissions (CH_4_ emission for paddies and uptake for upland) affected by Org-M compared to Non-F (**a**,**c**,**e**) and Min-F (**b**,**d**,**f**). The influence factors included climate types, soil pH, SOM and TN content. NTM means temperate monsoon climate; NTC means temperate continental climate; STM means subtropical monsoon climate. Numbers near right border are the numbers of comparisons. *P* < 0.05 when error bars do not overlap zero.

In general, Org-M significantly enhanced N_2_O emission compared to Non-F but decreased N_2_O emission relative to Min-F. In addition, the impact magnitude of Org-M was influenced by climate types, soil acidity, soil TN and soil organic matter content (Fig. 5a and b). For instance, Org-M significantly increased N_2_O emission (by 81% in paddy soils and 18% in upland soils) compared to Non-F but decreased the emission (by 14% in paddy soils and 11% in upland soils) compared to Min-F in the temperate monsoon climate. In the temperate continental monsoon climate, the effect of Org-M in comparison to Non-F and Min-F differed with land use, where N_2_O emission was increased in paddy soils but decreased in upland soils. Moreover, the greatest response of soil N_2_O emission to Org-M was found in upland soils (*RR*_++_ = 2.09 in comparison to Non-F and 0.69 in comparison to Min-F) in the subtropical monsoon climate but the smallest response was in paddy soils (*RR*_++_ = −0.17 in comparison to Min-F) under the same climate.

Clearly, there was more N_2_O emission from upland acid soils (pH < 7.0) than from upland alkaline soils (pH > 7.0). Specifically, *RR*_++_ of soil N_2_O emission from the acid soils was 1.4 (Org-M vs. Non-F) to 6.9 times (Org-M vs. Min-F) higher than those from the alkaline soils. For paddy soils, on the contrary, the *RR*_++_ from acid soils was smaller than those from alkaline soils for the groups.

Org-M significantly increased N_2_O emission compared to Non-F at different TN levels, and the largest difference between them was found when soil total N content ranged from 1.0 to 2.0 g kg^−1^ in both upland and paddy soils (Fig. 5a and b). In comparison with Min-F, soil N_2_O emission from Org-M increased in upland soils but decreased in paddy soils when TN ranged from 0.5 to 2.0 g kg^−1^. The largest response of soil N_2_O emission to Org-M vs. Min-F was found when TN was ≤0.5 in upland (−44%) or in the range of 0.5–1.0 g kg^−1^ in paddy soils (−72%).

The response of N_2_O emission to Org-M vs. Non-F or Min-F was also influenced by SOM content (Fig. 5a and b). In comparison with Non-F, Org-M significantly increased N_2_O emission by 205–460% in upland soils regardless of SOM content, but it slightly changed N_2_O emission (from −13% to 7%) in paddy soils when SOM ranged from 10 to 35 g kg^−1^. In comparison with Min-F, Org-M decreased soil N_2_O emission by 5–16% in paddy soils but increased the emission by 18–64% in upland soils when SOM was greater than 10 g kg^−1^. However, when SOM at a lower level (≤10 g kg^−1^), Org-M decreased N_2_O emission by 19% in upland soils compared with Min-F.

#### CO_2_ emission

In general, Org-M significantly increased soil CO_2_ emission compared to Non-F under all environmental conditions (climate type, soil pH, soil TN and SOM) and increased soil CO_2_ emission compared to Min-F under most of the conditions (Fig. 5c and d). The response of soil CO_2_ emission to Org-M was significantly affected by climate. For upland and paddy soils, CO_2_ emission increased by manure application compared to Min-F in all different climates except in temperate monsoon climate. Specifically, the greatest increase of CO_2_ emission rates occurred in the subtropical monsoon climate in both upland soils (with *RR*_++_ of 2.26) and paddy soils (with *RR*_++_ of 0.70). The CO_2_ emission decreased in paddy soils (with *RR*_++_ of −0.17) after manure application compared to Min-F.

The CO_2_ emission rate from Org-M increased in both upland and paddy soils in comparison with Non-F and in upland soils only in comparison with Min-F. In comparison with Min-F, Org-M did not significantly change CO_2_ emission in any paddy soil but for upland soils Org-M significantly increased CO_2_ emission by 52% in acid soils (pH ≤ 7.0) and by 10% in alkaline soils (pH > 7.0).

The results also showed that Org-M significantly increased soil CO_2_ emission (by 31–125%) in both upland and paddy soils compared with Non-F across the range of TN studied. However, Org-M decreased the emission compared to Min-F, when TN was less than 1.0 g kg^−1^. Notably, the effect sizes of *RR*_++_ in paddy soils were somewhat smaller than those in upland soils for all TN levels.

When SOM content was at 10–21 g kg^−1^ and ≥35 g kg^−1^, the datasets in paddy soils were not enough to achieve the meta-analysis. The effects of Org-M on soil CO_2_ emission (for both in comparison with Non-F and Min-F) were negatively correlated with SOM content except when SOM content was less than 10.0 g kg^−1^ in upland soils, which indicated that the decrement of SOM content could lead to the increment compared to Non-F and Min-F. In addition, the largest increment of soil CO_2_ emission in comparison with Min-F can be found when SOM content ranged from 10.0 to 21.0 g kg^−1^, respectively.

#### CH_4_ emission

In paddy soils, CH_4_ emission induced by Org-M varied with climate regions compared with Non-F and Min-F (Fig. 5e and f). In comparison to Min-F, Org-M increased CH_4_ emission by 89% and 71% in temperate monsoon climate and subtropical monsoon climate, respectively. In addition, Org-M increased CH_4_ emission by 122% in alkaline soils (pH > 7.0) and 104% in acid soils (pH ≤ 7.0) compared with Non-F. On the contrary, Org-M decreased CH_4_ emission by 36% in alkaline (pH > 7.0) and by 50% in acid soils (pH ≤ 7.0) compared to Min-F. In paddy soils, Org-M increased soil CH_4_ emission by 89–196% compared to Non-F and by 65–149% compared to Min-F at different soil TN levels. The response of soil CH_4_ emission to Org-M vs. Non-F was relatively small when SOM content ranged from 21 to 35 g kg^−1^, and was the largest at the content of SOM ≤ 21 g kg^−1^. In comparison to Min-F, Org-M increased soil CH_4_ emission in paddy soils by 60% to 116%, with the largest increase found when SOM content was ≤21 g kg^−1^.

In upland soils, after manure application soil CH_4_ uptake in the temperate monsoon climate were 2.9 and 2.6 times smaller than those in subtropical monsoon climate compared to Non-F and Min-F, respectively. In comparison to Non-F, Org-M increased soil CH_4_ uptake (by 86%) in acid upland soils but decreased in alkaline upland soils (by −44%). Similarly, the increment of CH_4_ uptake by Org-M over Min-F was 45% in acid upland soils and 12% in alkaline upland soils. At a lower TN level (≤1.0 g kg^−1^), Org-M decreased soil CH_4_ uptake by 51% and 20% compared to Non-F and Min-F, respectively. On the contrary, CH_4_ uptake was increased by Org-M by 83% and 43%, respectively, when TN ranged from 1.0 to 2.0 g kg^−1^. When SOM content was greater than 21 g kg^−1^, Org-M increased the CH_4_ uptake by 85% compared with Non-F but only 6% in comparison with Min-F, but there was insufficient data to analyze the CH_4_ uptake when SOM content was greater than 21 g kg^−1^.

## Discussion

Despite that previous studies have investigated mechanisms of GHGs emissions from soils with manure and non-manure amendments at different scale^38, 44–46^, there has been lack of systematic meta-analysis of impacts of manure application on GHGs emissions under a large range of environmental and management conditions (e.g. land uses, soil properties and N input). Here, we systemically and quantitatively analyzed the effects of Org-M on soil-derived GHGs emissions compared with the effects of non-fertilizer or chemical fertilizers in the China’s agriculture, based on the best available data reported in 90 journal articles. The meta-analysis in our study enabled pairwise comparisons between Org-M and Min-F or Non-F. Furthermore, the comparison between previous studies and our results indicated that current study conducted a more comprehensive analysis on the possible factors (climate, SOC, TN, pH and N input) influencing N_2_O, CO_2_ and CH_4_ emissions (Table 1)^5, 18, 37–39, 47–49^.

**Table 1:** Comparison of this study with other Meta-analysis on the GHGs emission from the organic amended soils.

Reference	Study area	Number of literature	Types of GHGs	Influence factors of the GHGs emissions referred in literature				
				Climate	TN	SOC	pH	N input
Skinner *et al*., (2014)	Global	19	CO_2_, N_2_O, CH_4_	—	√	√	√	—
Mondelaers *et al*., (2009)	Developed countries	10	N_2_O, CH_4_	—	—	√	—	—
Gregorich *et al*., (2005)	Eastern Canada	41	CO_2_, N_2_O, CH_4_	√	**—**	**—**	**—**	**—**
Feng *et al*., (2013)	Rice cropping systems in China	24	N_2_O, CH_4_	—	**—**	**—**	**—**	**—**
Zhao *et al*., (2016)	Agricultural soils in China	39	N_2_O, CH_4_	—	—	—	√	√
Luo *et al*., (2006)	Terrestrial ecosystems in China	104	CO_2_	—	—	—	—	—
Jeffery *et al*., (2016)	Global	42	CH_4_	—	—	—	√	√
Song *et al*., (2016)	Global	61	CO_2_, N_2_O, CH_4_	—	—	—	—	—
This study	Agricultural soils in China	90	CO_2_, N_2_O, CH_4_	√	√	√	√	√

Note: “−” means the dataset was not included in the literature. “√” means the dataset was not included in the literature.

### N_2_O emission

Generally, across China’s agricultural land use types covered in the current analysis, less N_2_O was emitted from Org-M than from Min-F. This is because N in the synthetic fertilizers is much more bioavailable than that in animal manures^50–52^. It has been demonstrated that different fertilizer types may dramatically affect N_2_O emission from agricultural soils in opposite ways^11, 12^. We found that soil N_2_O emissions from OM treatments were 14% lower than those from Min-F, but the soil N_2_O emissions from OM + CF were 3% higher than those from Min-F generally. Furthermore, we also found that Org-M-derived soil N_2_O emission was significantly lower than Min-F-derived emission, even though a same amount of total N was applied to the field (Fig. 4). It may be due to the different C/N ratio of manure, the application timing (cold or warm season), and different land uses^53–56^.

The results showed that the increment of soil N_2_O emission in upland soils in subtropical monsoon climate was much higher than those in temperate climate regions for both group of Org-M vs. Non-F and Org-M vs. Min-F. The reason may be due to the higher precipitation and temperature in the subtropical monsoon climate that can increase the decomposition of manure and availability of carbon, which can activate soil microbial respiration, reduce oxygen availability and generate anaerobic sites where denitrification is intensified^57, 58^. In the current study, the relative effects of Org-M on soil N_2_O emission behaved differently in the acid and alkaline upland soils, for which Org-M derived N_2_O emission was lower than Min-F at soil pH > 7, but much higher at soil pH ≤ 7. It was proved that some acidic soils had extremely high N_2_O production after manure application^59^. The previous study also confirmed that soil N_2_O production can be inhibited in soil with a high pH (>7) due to nitrification, denitrification, or dissimilatory N_2_O reduction to NH_4_^+^ and formation of intermediate products^60^. Soil N_2_O emission were also mainly influenced by SOM and TN, which has been confirmed by previous studies^61, 62^. Decomposition of SOM activated soil microbial respiration, consumed oxygen in the soil, accelerated the formation of the anaerobic environment, and indirectly enhanced the soil denitrification. Studies have found that the mineralization process of organic matter will supply mineral nitrogen in soil, and further enhance the formation and emission of N_2_O^58^.

### CO_2_ emission

Soil CO_2_ fluxes are mainly produced by soil microorganism and plant root respiration^63^. Our results revealed that Org-M significantly increased soil CO_2_ emission by 84% and 15% compared to Non-F and Min-F, respectively. It was reported that cumulative CO_2_ emission during crop growing seasons were 988 and 1130 g CO_2_m^2^ under manure applied with applications rate of 7500 and 22500 kg ha^−1^, respectively, which were 42 and 63% higher than the emissions from the no fertilization^16^. In addition, Org-M significantly stimulated CO_2_ emission by 23% compared with Min-F in upland soils, because addition of Org-M sharply increased soil organic C, particularly light fraction organic C that is more readily for microorganisms in respiration^64, 65^. Furthermore, OM and OM + CF led to 15% and 23% more CO_2_ emission than Min-F (*P* < 0.05), which was supported by a previous report that OM and OM + CF increased CO_2_ emission by 12% and 16% compared to Min-F in upland soils^14^. In a review, Qiao *et al*.^66^ concluded that application of manure combined with chemical fertilizer accelerated soil CO_2_ emission (555 g C m^−2^) by 27% more than chemical fertilizers alone (435 g C m^−2^) during the maize growing season. Elsewhere, Chen *et al*.^67^ found that repeated applications of manure in rice fields may reduce the increment of CO_2_ concentration compared to chemical fertilizer.

In addition, for all the studies in our database with same amount of N inputs between Org-M and Min-F, Org-M increased CO_2_ emission by 19% compared to Min-F generally (−36% in paddy soils and 26% in upland soils). It has been reported that in a same amount of total N input experiment, 40%, 60%, 80% and 100% of total N rate as organic N from manure, CO_2_ release were 0.9124, 0.6524, 0.4016 and 0.5132 t ha^−1^ yr^−1^ in paddy soils^68^, indicating Org-M can reduce the cumulative soil CO_2_ emission compared to Min-F^14^. However, some studies showed that manure combined with chemical N had no effects^69, 70^ or even increase CO_2_ emission from soils^71, 72^. Our results clearly demonstrated that manure could increase CO_2_ emission in upland soils but reduced the emission in paddy soils across China’s agricultural regions. However, the agricultural practices such as replacing Min-F with Org-M, decreasing the total N input rates and especially optimizing the ratio of Org-M in combination with Min-F need to be further investigated.

Soil CO_2_ emission clearly varied at different climate regions compared to Non-F and Min-F regardless upland or paddies. For instance, the differences of soil CO_2_ emission between Org-M and Min-F or Non-F at subtropical monsoon climate region were much higher than those at other climate regions (Fig. 5c and d). Prevalence of warm temperature in this climate type accelerates decomposition of organic C from soil pools or external sources and leads to C loss to the atmosphere. However, temperate climate regions with somewhat low temperature and precipitation have the less living biomass and the decomposition rate of organic C is often slow^14, 73–75^. It also showed that in comparison with Min-F, the increment of CO_2_ emission by Org-M in acid soils (pH ≤ 7.0) was larger than that in alkaline soils (pH > 7.0) regardless of upland or paddy soils, because Org-M usually increases soil pH and subsequently increases the solubility of CO_2_ and the formation of bicarbonate acid^76^, leading to a reduction in CO_2_ emission, especially in paddy fields. Soil N content can significantly increase the biomass of crops and then the carbon from plant root and residue can be increased, and the microbial activity can also be further promoted^77^. The results also showed that soil CO_2_ emission obviously varied with SOM content. It has been reported that the loss ratios of organic C input to the soils with low SOM content were more than those to the soils with high SOM content^78^.

### CH_4_ emission

It has been indicated that manure amendments may improve soil aeration, and thus decrease CH_4_ production and/or increase CH_4_ oxidation^79^, which can explain the greater net uptake of CH_4_ in the presence of Org-M in the upland field in the current study (Fig. 2). The flooding irrigation in paddy soils creates anaerobic conditions, which promotes the methanogens, increases CH_4_ emission, and enhances the activity of specific methane and ammonium oxidizing bacteria^29^. Generally, CH_4_ is the dominant gaseous product of anaerobic decomposition of organic matter, especially in an anoxic habitat as under paddy soils, which can explain the results from the current study that Org-M induced 111% and 72% more of CH_4_ emission compared with Non-F and Min-F in paddy soils (Fig. 2).

Previously, ammonium-based fertilizer was reported to inhibit CH_4_ oxidation in paddies, whereas the application of manure that contained more N than the fertilizer had no inhibitory effects^80^. Similar trends have been reported that different manure combined with chemical fertilizer significantly (P < 0.01) increased CH_4_ emission^81^. It has been reported that the cumulative CH_4_ emission from the pig manure plus chemical N fertilizer (50% chemical N + 50% N from manure) were 43% higher than that from the treatment of 100% chemical N fertilizer during rice growing seasons^82^. This might be because manure application enhanced soil microbial biomass and activity, which would promote CH_4_ production, and also suppress the activity of the relevant enzymes for microbial methane oxidation^24, 83^. In addition, Org-M increased 84% of CH_4_ emission compared to Min-F at the same amount N input, which fall within a range of 25–115% presented by previous reports in paddy soils^56, 81, 84^.

Compared with Non-F and Min-F, the increase of CH_4_ emission by Org-M were lower in temperate monsoon climate than those in subtropical monsoon climate (Fig. 5e and f), because the precipitation and temperature in temperate monsoon were more moderate than those in subtropical monsoon climate. Low temperatures can suppress microbial activities and metabolism and therefore production of CH_4_^85–87^. It was confirmed that high temperature induced high ventilation rates correlating to high CH_4_ emission in subtropical monsoon climate^88^. Soil pH also affects the soil CH_4_ emission, which could be significantly reduced with decrement of soil pH in paddies, and CH_4_ uptake could be significantly enhanced in upland soils by Org-M compared to Min-F when soil pH decreased (Fig. 5f). Our results systemically illustrated the response of CH_4_ emission to Org-M compared with Non-F and Min-F under different soil pH level for China’s agriculture, although it has been reported that soil CH_4_ uptake could be significantly enhanced by soil pH under the other farming practices (e.g., tillage and non-tillage) by other meta-analysis^5, 38^.

In the current study, the response of CH_4_ to Org-M compared with Non-F and Min-F did not show the obvious patterns under different TN levels. Similarly, it has been reported that the total CH_4_ emission did not correlate with TN with a R^2^ = 0.154, *P* > 0.05, which was caused by the unavailability to microorganisms of a large portion of soil organic matter under submerged conditions in short time^89^. The increase of CH_4_ emission positively correlated with SOM content and the decomposition of native SOM^90^. These decomposition processes provide predominant substrates for methanogens and stimulate the growth of methanogenic archaea^91^. In turn, this activity promotes CH_4_ production^92^. However, it deserves attention that although we collected all the available data, it is still not sufficient for assessing methane fluxes, especially in upland agricultural soils, and to draw solid conclusions about a farming system’s impact on CH_4_ fluxes.

## Conclusions

Over all studies included in the current meta-analysis, Org-M significantly enhanced soil N_2_O emission compared with Non-F and reduced soil N_2_O emission compared with Min-F. Furthermore, Org-M significantly promoted CO_2_ emission in upland soils but not in paddy soils compared with Min-F, and Org-M significantly increased CH_4_ emissions in paddy soils and uptake in upland soils. Based on the meta-analysis, the stimulation or suppression of manure application on soil GHGs emissions considerably varied with climate types, land uses, soil pH, soil TN and content of SOM, indicating that these influential factors need to be fully considered to optimize fertilization strategies to minimize GHGs. Given same amount of N inputs between different fertilization treatments, manure application significantly reduced soil N_2_O emission by 3% and CO_2_ emission by 36%, but increased CH_4_ emission by 84% compared to Min-F in paddy soils. Our results also demonstrated that compared with Min-F, emissions of N_2_O and CO_2_ from the soils with OM were all less than those from the soils with OM + CF. In addition, Org-M induced less soil N_2_O, CO_2_ emissions and more CH_4_ uptake compared with Min-F, under specific conditions such as temperate monsoon climate, alkaline soils and total N ranging from 0.5 to 1.0 g kg^−1^. Thus, some strategies such as replacing Min-F with Org-M and reducing total N application rate need to be designed according to different conditions in China to mitigate GHGs emission. Finally, responses of GHGs emissions to manure applied in agricultural soils as revealed by our analysis can be potentially useful for validating soil processed models and filling the gaps that lack of comparative studies on agricultural soil-derived GHGs emissions across China.

## Electronic supplementary material

Table S1
