# Supplementary material for: A synthetic analysis of greenhouse gas emissions from manure amended agricultural soils in China
Source: Sci Rep. 2017 Aug 14;7:8123. doi: 10.1038/s41598-017-07793-6 (PMC5556094; doi:10.1038/s41598-017-07793-6)
Supplement: Supplementary file 1 — Table S1 [file 41598_2017_7793_MOESM1_ESM.doc]

***Auxiliary material for***

**A synthetic analysis of greenhouse gas emissions from manure amended agricultural soils in China**

Fengling Ren^1^, Xubo Zhang^2^*, Jian Liu^3^, Nan Sun^1^*, Lianhai Wu^4^, Zhongfang Li^5^, Minggang Xu^1^

^1^ *Ministry of Agriculture Key Laboratory of Crop Nutrition and Fertilization,* *Institute of Agricultural Resources and Regional Planning, Chinese Academy of Agricultural Sciences, Beijing 100081, China.*

^2^ *Key Lab. of Ecosystem Network Observation and Modeling, Institute of Geographic Sciences and Natural Resources Research, Chinese Academy of Sciences, Beijing 100101, China*

*^3^ Department of Plant Science, Pennsylvania State University, University Park, Pennsylvania, 16802, USA.*

*^4^ Sustainable Soils and Grassland Systems Department, Rothamsted Research, North Wyke, Okehampton, Devon EX20 2SB, UK.*

*^5^Chemistry and Bioengineering College, Hezhou University, Hezhou 542899, China*

^*^ Corresponding author:

[zhangxb@igsnrr.ac.cn](mailto:zhangxb@igsnrr.ac.cn) (Xubo Zhang), Tel.: +86 10 64889523

[sunnan@caas.cn](mailto:sunnan@caas.cn) (Nan Sun), Tel.: +86 10 82105062

**Supplementary table for Online Only**

**Table S1** A list of 90 publications from which data were collected for current study.

**Table S1.** A list of 90 publications from which data were collected for current study.

| **No.** | **Reference** | **Location** | **MAT**  **(mm)** | **MAP (mm)** | **Land use** |
| --- | --- | --- | --- | --- | --- |
| 1 | Cai et al(2013) | 35°00'N, 114°24'E | 13.9 | 615 | U |
| 2 | Cai et al(2012) | 35°00'N, 114°24'E | 13.9 | 615 | U |
| 3 | Chen et al(2006) | 28°41'N, 121°26'E | 17.5 | 1926.7 | P |
| 4 | Chen et al(2012) | 39°44'N, 116°20'E | 11.6 | 556 | U |
| 5 | Chen et al(2014) | 47°26'N, 126°38'E | 1.5 | 550 | U |
| 6 | Ding et al(2003) | 35°03’N, 114°25’E | 13.9 | 615 | U |
| 7 | Ding et al(2007) | 35°00’N, 114°24‘E | 13.9 | 615 | U |
| 8 | Ding et al(2012) | 35°00’N, 114°24‘E | 13.9 | 615 | U |
| 9 | Dong et al(2005) | 36°57'N, 116°36'E | 13.2 | 538 | U |
| 10 | Dong et al(2005) | 36°57'N, 116°36'E | 13.2 | 538 | U |
| 11 | Gao et al(2009) | 32°12'N, 107°40'E | 9.1 | 578.5 | U |
| 12 | Guo et al(2016) | 31°02’N, 112°19’E | 16 | 1085 | P |
| 13 | Guo et al(2015) | 31°01'N, 112°12'E | 16.5 | 1085 | P |
| 14 | Hou et al(2012) | 28°08’N, 113°12’E | 17.1 | 1500 | P |
| 15 | Hu et al(2013) | 36°52'N, 115°10'E | 13.1 | 556.2 | U |
| 16 | Huang et al (2011) | 31°26'N, 119°47'E | 16 | 1100 | U-P |
| 17 | Huang et al(2009) | 26°45'N, 111°52'E | 18.1 | 1445 | U |
| 18 | Huang et al(2011) | 26°45’N, 111°52’E | 18.1 | 1445 | U |
| 19 | Huo et al(2013) | 28°08’N, 113°12'E | 17.1 | 1500 | P |
| 20 | Li et al(2013) | 26°45’N, 111°52’E | 17 | 1392.62 | P |
| 21 | Li et al(2013) | 28°68'N, 112°87'E | 17 | 1392.62 | P |
| 22 | Li et al(2009) | 35°12'N, 107°40'E | 9.1 | 578.5 | U |
| 23 | Li et al(2009) | 47°26'N, 126°38'E | 1.5 | 550 | U |
| 24-1 | Li et al(2009) | 31°05'N, 120°46'E | 15.7 | 1100 | P |
| 24-2 | Li et al(2009 | 116°20'N 28°15'E | 18.1 | 1400 | p |
| 25 | Li et al(2013) | 47°26'N, 126°38'E | 1.5 | 550 | U |
| 26 | Li et al(2013) | 47°26'N, 126°38'E | 1.5 | 550 | U |
| 27 | Li et al(2009) | 26°45’N, 111°52’E | 18.1 | 1445 | U |
| 28 | Li et al(2015) | 37°20'N, 116°38'E | 12.9 | 547.5 | U |
| 29 | Liang et al(2011) | 29°31'N, 106°16'E | 18.4 | 1105 | U-P |
| 30 | Liang et al(2013) | 120°40'E, 30°50'N | 15.7 | 1200 | P |
| 31 | Liang et al(2012) | 47°6'N, 126°8'E | 1.5 | 550 | U |
| 32 | Lin et al(2014) | 26°48′N, 119°18′E | 20 | 1359 | P |
| 33 | Liu et al(2016) | 42°31'N, 130°13'E | 4 | 475 | P |
| 34 | Liu et al(2013) | 36°51'N-37°06'N  117°50'E-118°10'E | 12.7 | 640.5 | U |
| 35 | Liu et al(2010) | 35°16'N, 107°30'E | 8 | 540 | U |
| 36 | Liu et al(2016) | 31°27'N, 120°25'E | 15.7 | 1100 | U-P |
| 37 | Liu et al(2009) | 38°52'N, 115°28E | 13 | 532 | U |
| 38 | Liu et al(2015) | 35°26'N, 117°50'E | 13.2 | 770.2 | U |
| 39 | Liu et al(2009) | 28°21’N, 116°10’E | 18.1 | 1537 | P |
| 40 | Liu et al(2014) | 31°16’N, 105°27’E | 17.3 | 826 | U |
| 41 | Liu et al(2009) | 31°05’N, 20°46’E | 15.7 | 1100 | P |
| 42 | Liu et al(2011) | 31°05’N, 20°46’E | 15.7 | 1100 | P |
| 43-1 | Liu et al(2013) | 28°22’N, 112°48’E | 17 | 1300 | P |
| 43-2 | Liu et al(2013) | 28°37’N, 116°26’E | 17.7 | 1400 | P |
| 43-3 | Liu et al(2013) | 31°10’N, 120°38’E | 18.3 | 1100 | P |
| 43-4 | Liu et al(2013) | 31°23’N, 120°38’E | 18.3 | 1100 | P |
| 43-5 | Liu et al(2013) | 30°31’N, 105°35’E | 17.4 | 930 | P |
| 43-6 | Liu et al(2013) | 26°48’N, 119°18’E | 19.5 | 1350 | P |
| 44 | Liu et al(2016) | 31°16’N, 105°27’E | 17.3 | 836 | U |
| 45 | Long et al(2014) | 28°15'N, 116°55'E | 17.6 | 1795 | U |
| 46 | Lu et al(2015) | 39°57'N-40°27'N  118°6'E-118°37'E | 10.1 | 817 | U |
| 47 | Meng et al(2008) | 35°00’N, 114°24‘E | 13.9 | 605 | U |
| 48 | Meng et al(2008) | 35°00’N, 114°24‘E | 13.9 | 615 | U |
| 49 | Meng et al(2005) | 35°00’N, 114°24‘E | 13.9 | 615 | U |
| 50 | Meng et al(2005) | 35°04’N, 113°10‘E | 13.9 | 615 | U |
| 51 | Qiao et al(2014) | 47°26'N, 126°38'E | 1.5 | 550 | U |
| 52 | Qiao et al(2007) | 47°27'N, 126°55'E | 1.5 | 550 | U |
| 53 | Qiao et al(2012) | 47°27'N, 126°55'E | 1.5 | 550 | U |
| 54 | Qin et al(2006) | 27°58'N, 112°36'E | 17.5 | 1350 | P |
| 55 | Shi et al(2013) | 47°26’N, 126°38’E | 1.5 | 550 | U |
| 56 | Shi et al(2011) | 35°12'N, 107°40'E | 9.1 | 578.5 | U |
| 57 | Shi et al(2011) | 28°08’N, 113°12’E | 17.1 | 1316 | P |
| 58 | Shi et al(2010) | 28°08’N, 113°12’E | 17.1 | 1500 | P |
| 59 | Song et al(1997) | 37°53'N, 14°14'E | 12.8 | 474 | U |
| 60 | Sun et al(2012) | 32°29'N, 118°36'E | 15.3 | 970 | U-P |
| 61 | Sun et al(2012) | 32°29'N, 118°36'E | 15.3 | 970 | U-P |
| 62 | Tong et al(2011) | 26°45'N, 111°52'E | 4.5 | 525 | U |
| 63 | Wang et al(2014) | 28°33'N, 113°19'E | 17 | 1370 | P |
| 64 | Wang et al(2013) | 31°33'N, 120°43'E | 16.6 | 1178 | U-P |
| 65 | Wei et al(2010) | 35°12’N, 107°40’E | 9.1 | 585 | U |
| 66 | Wu et al(2011) | 28°08'N, 113°12'E | 17.1 | 1500 | P |
| 67 | Xie et al(2012) | 45°40’N, 126°35’E | 3.5 | 533 | U |
| 68 | Xin et al(2012) | 35°12'N-35°16'N  107°40'E-107°42'E | 9.1 | 578.5 | U |
| 69 | Xiong et al(2002) | 28°15’N, 116°55’E | 17.6 | 1795 | P |
| 70 | Xu et al(2008) | 34°17'N, 108°04'E | 14.9 | 525 | U |
| 71 | Xue et al(2010) | 32°12'N, 107°40'E | 9.1 | 578.5 | U |
| 72 | Yang et al(2015) | 31°32'N, 120°55'E | 17.73 | 1890 | U-P |
| 73 | Yang et al(2007) | 40°N, 120°’E | 8 | 714 | U |
| 74 | Yi et al(2006) | 28°41'N, 121°26'E | 17.5 | 1926.7 | P |
| 75 | Zeng et al(2012) | 30°52'N, 105°58E | 17.3 | 836 | U |
| 76 | Zhai et al(2011) | 26°45'N, 111°52'E | 18.6 | 1454 | U |
| 77 | Zhai et al(2013) | 40°12'N, 118°18'E | 10.1 | 804.2 | U |
| 78 | Zhai et al(2013) | 40°12'N 118°18'E | 10.1 | 804.2 | U |
| 79 | Zhang et al(2012) | 40°N 120°’E | 8.1 | 724.3 | P |
| 80 | Zhang et al(2013) | 35°19'N 113°51'E | 14 | 656.3 | U |
| 81 | Zhang et al(2011) | 38°8'N 106°16'E | 8.9 | 192.9 | U |
| 82 | Zhang et al(2012) | 35°04'N 113°10'E | 13.9 | 615 | U |
| 83 | Zhang et al(2012) | 38°71'N 115°15'E | 12.3 | 555 | U |
| 84 | Zhao et al(2014) | 31°15'N 121°12E | 15.6 | 1161.7 | U-P |
| 85 | Zheng et al(2007) | 31°05'N 120°46'E | 17.73 | 1890 | U |
| 86 | Zhou et al(2014) | 40°12'N 118°18'E | 10.2 | 804.1 | U |
| 87 | Zhou et al(2011) | 37°38’N 112°51’E | 9.1 | 489 | U |
| 88 | Zhu et al(2013) | 31°16'N 105°27'E | 17.3 | 826 | U |
| 89 | Zou et al(2006) | 31°52’N 118°50’E | 15.4 | 1106.5 | U |
| 90 | Zou et al(2003) | 32°04'N 118°78'E | 15.4 | 1106.5 | P |

**MAT**: Mean annual temperature (°C)

**MAP**: Mean annual precipitation (mm)

**Land use** types: U: Upland; P: Paddy; U-P: Upland -Paddy rotation.

**References：**

Cai Y J, Ding W X. Nitrous oxide emissions from Chinese maize–wheat rotation systems: A 3-year field measurement. Atmos Environ, 2013, 65(65):112–122.

Cai Y, Ding W, Luo J. Spatial variation of nitrous oxide emission between interrow soil and interrow plus row soil in a long-term maize cultivated sandy loam soil. Geoderma, 2012, s 181–182:2-10.

Chen Y, Wu C Y, et al. Emission and fixation of CO_2_ from soil system as influenced by long-term application of organic manure in paddy soils. J Integr Agr, 2006, 5(6):456-461. (In Chinese)

Chen Y X. Study on the ecological and environmental impacts of liquid digestate from swine farm applied on cropland. Scientia Agricultura Sinica, 2012. (In Chinese)

Chen Z, Ding W, Luo Y, et al. Nitrous oxide emissions from cultivated black soil: A case study in Northeast China and global estimates using empirical model. Global Biogeochem Cy, 2014, 28(11):1311–1326.

Ding H, Cai G, Wang Y, et al. Nitrification-denitrification loss and NO_2_ emission from maize-wheat rotation system in North China. J Agro-environ Sci, 2003. (In Chinese)

Ding W, Lei M, Yin Y, et al. CO_2_ emission in an intensively cultivated loam as affected by long-term application of organic manure and nitrogen fertilizer. Soil Biol Biochem, 2007, 39(2):669–679.

Ding W, Luo J, Li J, et al. Effect of long-term compost and inorganic fertilizer application on background N_2_O and fertilizer-induced N_2_O emissions from an intensively cultivated soil. Sci Total Environ, 2012, 465(6):115-124.

Dong Y H, Yun-Sheng L I, et al. Influence of fertilization and environmental Factors on CO_2_ and N_2_O fluxes from agricultural soil. J Agro-environ Sci, 2005. (In Chinese)

Dong Y. Effects of organic manures on CO_2_ and CH_~~4~~_ fluxes of farmland. Chin J Appl Ecol, 2005, 16(7). (In Chinese)

Gao H Y, Guo S L, Liu W Z, et al. Soil respiration and carbon fractions in winter wheat cropping system under fertilization practices in arid-highland of the Loess Plateau. Acta Ecologica Sinica, 2009. (In Chinese)

Guo T F. Effects of fertilizer managements on greenhouse gas emissions and nutrient status in paddy soil. J Plant Nutr, 2016, 22(2): 337-345. (In Chinese)

Guo T F. Effect of fertilization management on greenhouse gas emission and soil microbial properties in rice-wheat rotation system. Chinese Academy of Agricultural Sciences, 2015. 214.(In Chinese)

Hou X L. Study on soil carbon sequestration and carbon emission mitigation under different fertilization. Chinese Academy of Agricultural Sciences Master Dissertation, 2012. 143.(In Chinese)

Hu X K, Su F, Ju X T, et al. Greenhouse gas emissions from a wheat-maize double cropping system with different nitrogen fertilization regimes. Environ Pollut, 2013, 176(5):198-207.

Huang H Y, Cao J L, Jin H M, et al. Influence of application of digested pig slurry on nitrous oxide emission under rice-wheat rotation system. J Agro-Environ Sci, 2011, 30(11):2353-2361. (In Chinese)

Huang J, Liu H.B, Wang B R. CO_2_, N_2_O emission from red soil dry-land under long-term fertilization. Chin Agric Bull, 2009, 25(24):428-433. (In Chinese)

Huang J. CO_2_ and N_2_O emissions from red soil during wheat and corn growing seasons under different patterns of long-term fertilization. J Ecol Rural Environ. 2011, 27(4):7-13. (In Chinese)

Huo L J, Xiong H J I, Wu J M, et al. The effect of organic manures application on methane emission and its simulation in paddy fields. J Agro-Environ Sci, 2013, 32(10):2084-2092. (In Chinese)

Huo L J, et al. The effect of organic manures application on methane emission and its simulation in paddy fields. Acta Agricol Scien tica Sinica, 2013, 32 (10): 2084-2092. (In Chinese)

Li B, Rong X M, Xie G X, et al. Effect of combined application of organic and inorganic fertilizers on greenhouse gases exchange and comprehensive global warming potential in paddy fields. J Soil and Water Conserv, 2013, 27(6):298-304. (In Chinese)

Li B. Effect of combined application with organic and inorganic fertilizers on rice growth and greenhouse gas emission from double-cropping paddy fields. Hunan Agricultural University, 2013. (In Chinese)

Li F L, The effect of long-term fertilization on wheat yield and the ecological environment in the dry-land of loess plateau. Northwest A&F University, 2009. (In Chinese)

Li H B, Han X Z, Qiao Y F, et al. Carbon dioxide emission from black soil as influenced by land-use change and long-term fertilization. Commun Soil Sci Plan, 2009, 40(7-8):1350-1368.

Li J J. Net carbon sink effect and cost/benefit evaluation in rice-rape rotation system in Tai Lake region and rice-rice system in red soil region in Jiangxi under long-term fertilization. Nanjing Agricultural University, 2009. (In Chinese)

Li L J, Han X Z, You M Y, et al. Nitrous oxide emissions from Mollisols as affected by long-term applications of organic amendments and chemical fertilizers. Sci Total Environ, 2013, 452-453C (5):302-308.

Li L J, You M Y, Ding X L, et al. Soil CO_2_ emissions from a cultivated Mollisol: effects of organic amendments, soil temperature, and moisture. Eur J Soil Biol, 2013, 55(3):83-90.

Li M Y, Xu M G, Wang B R, et al. Effect of long-term fertilizations on N_2_O emission and its relationship with red soil properties in southern China. J Agro-Environ Sci, 2009:2645-2650. (In Chinese)

LI Y Q, Tang J W, et al. Effect of organic and inorganic fertilizer on the emission of CO_2_ and N_2_O from the summer maize field in the north China plain. Scientia Agricultura Sinica, 2015, 48(21): 4381-4389. (In Chinese)

Liang H. Effect of different fertilization on CO_2_ emission for a paddy-upland rotation purple soil under wheat. Southwest University, 2011. (In Chinese)

Liang X Q, Li H, Wang S X, et al. Nitrogen management to reduce yield-scaled global warming potential in rice. Field Crop Res, 2013, 146(3):66-74.

Liang Xin, Effects of long-term fertilization on soil nitrification and N_2_O emission in the semiarid loess plateau. Northwest A&F University, 2012. (In Chinese)

Liang Y, Han X Z, Qiao Y F, et al. Soil respiration and carbon budget in black soils of wheat-maize-soybean rotation system. Chin J Eco-Agric, 2012, 20(4):395-401. (In Chinese)

Lin C, Wang F, Chun-Mei H E, et al. CH_4_ emission characteristics of Yellow-mud field under long-term fertilization in southern China and its greenhouse effect. Hunan Agric Sci, 2014. (In Chinese)

Liu C H, Fu M J, Wu F R, et al. Effect of different fertilizer types on emissions of greenhouse gas in the Northern paddy. Hubei Agric Sci, 2016. (In Chinese)

Liu D X, Optimal fertilization reduced greenhouse gas emission of wheat-maize cropping system. Shandong Agricultural University, 2013. (In Chinese)

Liu E, Yan C, Mei X, et al. Long-term effect of chemical fertilizer, straw, and manure on soil chemical and biological properties in northwest China. Geoderma, 2010, 158(3–4):173-180.

Liu H J, et al. Effects of different combined application ratio of organic-inorganic fertilization on CH_4_ and N_2_O emissions in paddy season. Ecol Environ, 2016, 25(5): 808-814. (In Chinese)

Liu H M. Release of carbon dioxide and its mechanism in summer maize and winter wheat rotation. Agricultural University of Hebei Province, 2009. (In Chinese)

Liu H, Li J, Li X, et al. Mitigating greenhouse gas emissions through replacement of chemical fertilizer with organic manure in a temperate farmland. Science Bulletin, 2015, 60(6):598-606.

Liu M, Feng H, Chen X, et al. Organic amendments with reduced chemical fertilizer promote soil microbial development and nutrient availability in a subtropical paddy field: The influence of quantity, type and application time of organic amendments. Appl Soil Ecol, 2009, 42(2):166-175.

Liu W L, et al. Effects of fertilizer application regimes on soil N_2_O emissions in the croplands of purple soil in the Sichuan Basin during wheat season. Chin J Eco-Agric, 2014, 22(9):1029-1037. (In Chinese)

Liu X Y, et al. CO_2_ emission under long-term different fertilization during rape growth season of a paddy soil from Tai Lake region, China. J Agro-Environ Sci, 2009. (In Chinese)

Liu X Y, et al. Greenhouse gas emission and C intensity for a long-term fertilization rice paddy in Tai Lake region, China. J Agro-Environ Sci, 2011, 30(9):1783-1790. (In Chinese)

Liu X Y. Effects of organic materials on carbon sequestration and productivity of farmland: field experiment and meta-analysis. Nanjing Agricultural University, 2013. (In Chinese)

Liu Y, et al. Effect of fertilization regime on soil N_2_O emission from upland field under wheat-maize rotation system. Acta Pedologica Sinica,2016,53(3):735-745. (In Chinese)

Long G Q, Jiang Y J, Sun B. Seasonal and inter-annual variation of leaching of dissolved organic carbon and nitrogen under long-term manure application in an acidic clay soil in subtropical China. Soil & Tillage Research, 2014, 146:270–278.

Lu Tao, Effect of different carbon and nitrogen management practices on N_2_O emissions from spring maize field. Shihezi University, 2015. (In Chinese)

Meng L, Cai Z C. Effects of long-term fertilization on N distribution and N_2_O emission in fluvo-aquci soil in North China. Acta Ecologica Sinica, 2008, 28(12):6197-6203. (In Chinese)

Meng L, Ding W X, He Q X, et al. Effect of long-term fertilization on soil respiration flux and its components under winter wheat/summer maize rotation. Soils, 2008, 40(5):725-731. (In Chinese)

Meng L, Ding W X, He Q X, et al. Effects of long-term fertilization on N partitioning and N_2_O emission in typical aquatic soils of North China. Acta Ecologica Sinica, 2008, 28 (12): 6197-6203. (In Chinese)

Meng L, Ding W, Cai Z. Long-term application of organic manure and nitrogen fertilizer on N_2_O emissions, soil quality and crop production in a sandy loam soil. Soil Biol Biochem, 2005, 37(11):2037-2045.

Meng L, Sheng W. Storage of soil organic C and soil respiration as effected by long-term quantitative fertilization. Adv Earth Sci, 2005,20(6):687-692. (In Chinese)

Qiao Y F, Han X Z, Doane T A, et al. Emission of CO_2_ and N_2_O from maize-soybean rotations under five long-term fertilizer regimes in northeastern China. J Food Agriculture Environ, 2014, 12(2):492-497.

Qiao Y F, Miao S J. Soil respiration affected by fertilization in black soil. Acta Pedologica Sinica,2007,44(6):1028-1035. (In Chinese)

Qiao Y F. The carbon and nitrogen conversion process affected by fertilization in Chinese Mollisols. Jilin Agricultural University, 2012. (In Chinese)

Qin X B, Li Y E, et al. Methane and nitrous oxide emission from paddy field under different fertilization treatments. Transactions of the Chinese Society of Agricultural Engineering, 2006, 22(7):143-148.

Shi H G. Impacts of management regimes on soil N_2_O emission from Mollisols in agroecosystem. Northeast Agricultural University, 2013. (In Chinese)

Shi Pei. Effect of long-term application of fertilization on wheat yield and emission of the CO_2_ and N_2_O from soil in loess Plateau. Northwest A&F University, 2011. (In Chinese)

Shi S W, L Y E, Li M D, et al. Annual CH_4_ and N_2_O emissions from double rice cropping systems under various fertilizer regimes in Hunan Province, China. Chin J Atmosph Sci, 2011,35(4):707-720. (In Chinese)

Shi S W. Mitigation option of CH_4_ and N_2_O emission from rice fields. Chinese Academy of Agricultural Sciences Master Dissertation, 2010. (In Chinese)

Song W Z, Wang S B, et al. Emission of nitrous oxide from dryland in northern China. Adv Environ Sci, 1997, 5(4):49-55. (In Chinese)

Sun G F, Zheng J C, Chen L G, et al. Effects of application of pig manure in combination with chemical fertilizers on CH_4_ and N_2_O emissions and their greenhouse effects in wheat field. J Ecol Rur Environ, 2012, 28(4):349-354. (In Chinese)

Sun G F, Zheng J C, Chen L G, et al. Effects of pig manure and biogas slurry application on CH_4_ and N_2_O emissions and their greenhouse effects on paddy field. Journal of China Agricultural University, 2012. (In Chinese)

Tong F, The feature of nitrogen component content and emission of N_2_O on the black soil under long-term fertilization. Jiangxi Agricultural University, 2011. (In Chinese) 166

Wang C, Shen J L, Zheng L, et al. Effects of combined applications of pig manure and chemical fertilizers on CH_4_ and N_2_O emissions and their global warming potentials in paddy fields with double-rice cropping. Environ Sci, 2014, 35(8):3120-7. (In Chinese)

Wang J, Chen Z, Ma Y, et al. Methane and nitrous oxide emissions as affected by organic–inorganic mixed fertilizer from a rice paddy in southeast China. J Soil Sediment, 2013, 13(8):1408-1417.

Wei X R, Hao M D, Xue X H, et al. Nitrous oxide emission from highland winter wheat field after long-term fertilization. Biogeosciences Discussions, 2010, 7(10):4539-4563.

Wu J M, et al. The Effect of different organic manures treatments on methane emission from single-cropping paddy fields. Acta Agriculata Scientia Sinica, 2011, 30 (8): 1688-1694. (In Chinese)

Xie L Y, Ye D D, Guo L P, et al. Effects of different fertilization modes on soil greenhouse gases emission from black soil in northeast China. Annual meeting of China Meteorological Society. 2012. (In Chinese)

Xin L. Long-term fertilization on soil nitrification and N_2_O emission in the Loess Plateau. Northwest A & F University, 2012. (In Chinese)

Xiong Z Q, Xing G X, Tsuruta H, et al. Measurement of nitrous oxide emissions from two rice-based cropping systems in China. Nutr Cycl Agroecos, 2002, 64(1):125-133.

Xu J, A Study of Fertilization and nitrous oxide losses on loess soil. Northwest A&F University, 2008. (In Chinese)

Xue X H, Effect of fertilization on nitrate leaching and greenhouse gases emission in the typical dry-farming area. University of Chinese Academy of Sciences, 2010. (In Chinese)

Yang B, Xiong Z, Wang J, et al. Mitigating net global warming potential and greenhouse gas intensities by substituting chemical nitrogen fertilizers with organic fertilization strategies in rice–wheat annual rotation systems in China: A 3-year field experiment. Ecological Engineering, 2015, 81:289-297.

Yang J, Han X, Zhan X, et al. Effects of different fertilization on N_2_O emission in brown field. Ecol Environ, 2007, 16(2):560-563. (In Chinese)

Zeng Z B. N_2_O emission characteristics of different fertilization treatments in wheat-maize rotation in hilly area of purple soil. J Anhui Agric Sci, 2012. (In Chinese)

Zhai L M, Liu H B, et al. Long-term application of organic manure and mineral fertilizer on N_2_O and CO_2_, emissions in a red soil from cultivated maize-wheat rotation in China. J Integr Agr, 2011, 10(11):1748-1757.

Zhai Z, Wang L, Li H, et al. Nitrous oxide emissions and net greenhouse effect from spring-maize field as influenced by combined application of manure and inorganic fertilizer. J Agro Environ Sci, 2013, 32(12):2502-2510. (In Chinese)

Zhai Z. Studies on N_2_O emission and mitigation of spring maize field in north of China—A case study from Qian xi county, in eastern Yan shan mountain. Chinese Academy of Agricultural Sciences, 2013. (In Chinese)

Zhang H, Dong H, et al. Influence of organic fertilization on N_2_O fluxes from corn field. Agricultural Science & Technology & Equipment, 2012. (In Chinese)

Zhang H, Guo L P, et al. Effects of different management measures on soil CO_2_ and N_2_O emission from winter wheat field in north China Plain. Chin J Soil Sci, 2013(3):653-659. (In Chinese)

Zhang H. Gaseous loss and balance of nitrogen from paddy field irrigation area of the upper Yellow River. Chinese Academy of Agricultural Sciences, 2011. (In Chinese)

Zhang J B, Zhu T B, Cai Z C, et al. Effects of long-term repeated mineral and organic fertilizer applications on soil nitrogen transformations. Eur J Soil Sci, 2012, 63(1):225–234.

Zhang Y, Liu J, Mu Y, et al. Nitrous oxide emissions from a maize field during two consecutive growing seasons in the north China plain. J Environ Sci, 2012, 24(1):160-168.

Zhao Z, Yue Y B, Zhang Y, et al. Impact of different fertilization practices on greenhouse gas emission from paddy field. J of Agro-Environ Sci, 2014. (In Chinese)

Zheng J, Zhang X, Li L, et al. Effect of long-term fertilization on C mineralization and production of CH_4_ and CO_2_ under anaerobic incubation from bulk samples and particle size fractions of a typical paddy soil. Agr Ecosyst Environ, 2007, 120(2–4):129-138.

Zhou M, Zhu B, Brüggemann N, et al. N_2_O and CH_4_ emissions, and NO_3_^−^ leaching on a crop-yield basis from a subtropical rain-fed wheat–maize rotation in response to different types of nitrogen fertilizer. Ecosystems, 2014, 17(2):286-301.

Zhou P, Yu E L, Liu L M, et al. Effects of fertilization and environment factors on N_2_O emission in spring corn field in north China Plain-A case study of Jin zhong in Shanxi province. Chin J Agrometeorol, 2011. (In Chinese)

Zhu Z B, Zhu B, Liu X M, et al. Effects of fertilization on N_2_O emission and denitrification in purple soil during summer maize season in the Sichuan Basin. Acta Pedologica Sinica,2013,50(1):130-137. (In Chinese)

Zou J W, Huang Y, Zong L G, et al. Effect of organic material incorporation in rice season on N_2_O emissions from following winter wheat growing season. Environ Sci, 2006, 27(7):1264-1268. (In Chinese)

Zou J, Huang Y, Zong L, et al. Integrated effect of incorporation with different organic manures on CH_4_ and N_2_O emissions from rice paddy. Chin J Environ Sci, 2003, 24(4):7-12. (In Chinese)
